# Decoding of the spike timing of primary afferents during voluntary arm movements in monkeys

**DOI:** 10.3389/fnins.2014.00097

**Published:** 2014-05-09

**Authors:** Tatsuya Umeda, Hidenori Watanabe, Masa-aki Sato, Mitsuo Kawato, Tadashi Isa, Yukio Nishimura

**Affiliations:** ^1^Department of Developmental Physiology, National Institute for Physiological Sciences, National Institutes of Natural SciencesOkazaki, Japan; ^2^Neural Information Analysis Laboratories, Advanced Telecommunications Research Institute InternationalKyoto, Japan; ^3^Computational Neuroscience Laboratories, Advanced Telecommunications Research Institute InternationalKyoto, Japan; ^4^Department of Physiological Sciences, School of Life Science, The Graduate University for Advanced Studies (SOKENDAI)Hayama, Japan; ^5^PRESTO, Japan Science and Technology AgencyKawaguchi, Japan

**Keywords:** dorsal root ganglion, proprioceptive interface, multichannel recording, integrate-and-fire model, brain machine interface

## Abstract

Understanding the mechanisms of encoding forelimb kinematics in the activity of peripheral afferents is essential for developing a somatosensory neuroprosthesis. To investigate whether the spike timing of dorsal root ganglion (DRG) neurons could be estimated from the forelimb kinematics of behaving monkeys, we implanted two multi-electrode arrays chronically in the DRGs at the level of the cervical segments in two monkeys. Neuronal activity during voluntary reach-to-grasp movements were recorded simultaneously with the trajectories of hand/arm movements, which were tracked in three-dimensional space using a motion capture system. Sixteen and 13 neurons, including muscle spindles, skin receptors, and tendon organ afferents, were recorded in the two monkeys, respectively. We were able to reconstruct forelimb joint kinematics from the temporal firing pattern of a subset of DRG neurons using sparse linear regression (SLiR) analysis, suggesting that DRG neuronal ensembles encoded information about joint kinematics. Furthermore, we estimated the spike timing of the DRG neuronal ensembles from joint kinematics using an integrate-and-fire model (IF) incorporating the SLiR algorithm. The temporal change of firing frequency of a subpopulation of neurons was reconstructed precisely from forelimb kinematics using the SLiR. The estimated firing pattern of the DRG neuronal ensembles encoded forelimb joint angles and velocities as precisely as the originally recorded neuronal activity. These results suggest that a simple model can be used to generate an accurate estimate of the spike timing of DRG neuronal ensembles from forelimb joint kinematics, and is useful for designing a proprioceptive decoder in a brain machine interface.

## Introduction

Researchers have developed a brain-machine interface (BMI) that allows patients or experimental animals to control a robotic arm by translating neural signals into control signals for the device (Hochberg et al., 2006, 2012; Velliste et al., 2008; Yanagisawa et al., 2012; Collinger et al., 2013). Furthermore, studies have shown that monkeys can use cortical activity to control functional electrical stimulation of muscles (Moritz et al., 2008; Ethier et al., 2012) and the spinal cord (Nishimura et al., 2013), and restore volitional control of the paretic hand. In these approaches, the control of a prosthetic device to a desired target has relied mainly on visual feedback for the position of the prosthesis. Since the ability to control hands and arms in a dexterous and compliant manner depends on somatosensory signals from the body (Ghez et al., 1990; Gentilucci et al., 1994; Gordon et al., 1995), somatosensory feedback should be provided for precise control of the prosthetic limb precisely and exploratorily (Biddiss and Chau, 2007). Recently a somatosensory BMI with a tactile feedback system, such as direct electrical stimulation of the primary sensory cortex, has been developed (O'Doherty et al., 2011; Tabot et al., 2013). Experimental animals perceived an electrical stimulation train as if it was a mechanical stimulus of the limbs at the corresponding frequency. Proprioceptive information can also be used to increase accuracy of prosthesis control (Johnson et al., 2013), but proprioceptive information has not been returned directly to the brain in the current frame of BMI research.

Primary sensory nerves are an appropriate site for the delivery of electrical stimulation to provide proprioceptive information to the subject. First, movements with a high number of degrees of freedom in three-dimensional space yield considerable positional information of a prosthetic device. Ensemble neural recordings in the dorsal root ganglia (DRGs) of anesthetized animals have shown that the activity of neuronal ensembles encoded high dimensional information of limb kinematics (Stein et al., 2004; Wagenaar et al., 2011; Weber et al., 2011; Umeda et al., 2012). Second, the sensation of limb movement may be induced artificially by the delivery of vibrations to a tendon (Goodwin et al., 1972; Craske, 1977; McCloskey et al., 1983; Roll et al., 2009; Thyrion and Roll, 2010) or the direct electrical stimulation of afferents (Gandevia, 1985; Dhillon and Horch, 2005). Individuals who felt the illusory movement of the stimulated hand could indicate the direction of the movement using the other hand. Thus, artificial kinesthetic sensations may allow individuals to adapt easily to such feedback signals containing considerable positional information of a prosthesis. The rigid link between limb movements, the neural activity of peripheral afferents, and kinesthetic sense suggests peripheral afferents as an appropriate site for a proprioceptive interface.

Estimating the neuronal firing pattern from the positional information of the upper limb is of obvious utility for the development of a sensory-motor BMI with a proprioceptive feedback system. The development of a motor BMI has stemmed from the establishment of decoding algorithms, which translate neuronal ensemble activity into limb movements and muscle activity (Chapin et al., 1999; Wessberg et al., 2000; Serruya et al., 2002; Morrow and Miller, 2003). Similarly, a decoding model would help to determine the optimal stimulus parameters to elicit artificial kinesthetic sensation. Spiking neuron models can reproduce the timing of spikes elicited by an external stimulus with high temporal precision (Gerstner and Kistler, 2002). An integrate-and-fire (IF) model is a simple phenomenological model of spike generation. Recent studies have demonstrated that various IF models accurately estimate spike timing, and describe some of the important physiological properties of the recorded sensory neurons (Pillow et al., 2005; Kim et al., 2010; Dong et al., 2013). For a model to have a practical use in an online BMI, it should be simple, with a small computational overhead. Furthermore, the reproduced spike patterns generated by the model should reliably encode the external stimulus that elicited the original neural firing pattern.

In the present study, we performed multichannel recordings from the cervical DRGs of awake monkeys during voluntary reach-to-grasp movements. First, we investigated whether a population of DRG neurons recorded from behaving monkeys could encode the forelimb joint kinematics. Next, we investigated whether the temporal firing pattern of DRG neurons can be reconstructed from forelimb joint kinematics using an IF model incorporating the sparse linear regression (SLiR) algorithm, which selects important input signals to reduce the computational time (Sato, 2001). Finally, we examined whether the reconstructed spike pattern contained positional information to validate the model.

## Materials and methods

One adult male monkey (Monkey T) and one adult female monkey (Monkey C) (*Macaca mulatta*) were used in the present study. The experiments were approved by the experimental animal committee of the National Institute of Natural Sciences of Japan (Approval Nos.: 10A203, 11A168, and 12A139) and were performed in accordance with the Weatherall report, “The use of non-human primates in research.” Before the experiments, the animals were housed individually on a 12-h light/dark cycle.

### Behavioral task

Monkey C was trained to perform a reach-to-grasp task with its right hand, as described previously (Shin et al., 2012). The object to grasp was a small plastic knob that was attached to the end of a joystick controller lever. To start a trial, the monkey placed its hand on a button located in front of a chair for 2–2.5 s. After a go cue was given as a beep sound, the monkey pulled the knob and then returned its hand to the button. When the monkey successfully pushed the button and pulled the knob to the required displacement of 6 cm, it received a reward. Monkey T was trained to perform a reach-to-grasp task with its right hand. The object was a small piece of potato. The monkey launched a trial by placing its hand in front of a chair, and then a piece of potato was presented in front of the monkey. The monkey was required to take the potato, eat it, and then return its hand to the original position. In the reach-to-grasp task, both the proximal and distal forelimbs were active. This task allowed us to analyze the kinematics from multiple joints of the forelimb. Monkey C performed the task for 5 sessions of 10 min each, in which the monkey conducted 136.8 ± 5.3 (mean ± standard deviation) trials per session. Monkey T performed the task for 1 session of 2 min, which contained 21 trials.

### Surgery

A mixture of xylazine (0.4 mg/kg; Bayer Health Care, Monheim, Germany) and ketamine (5 mg/kg; Daiichi Sankyo, Tokyo, Japan) was used to induce satisfactory sedation of the monkeys. Then, the monkeys were anesthetized with isoflurane (exhaled level; 1–2%) and nitrous oxide gas (33%). The monkeys were paralyzed using pancuronium bromide (Mioblock; 0.2 mg·kg^−1^·h^−1^; Schering-Plough Corporation, Kenilworth, NJ). Expiratory CO_2_ levels were maintained within the physiological range (3.3–4.2%). Assessment of the depth of anesthesia was done continuously by checking the stability of expiratory CO_2_ levels and the heart rate.

After shaving the hair on the back, the C3 through Th2 vertebrae were exposed bilaterally, and stainless screws were inserted into the lateral mass of each vertebra on the bilateral sides. After the lateral mass of the C5–Th1 segments was dissected on the right side, two multi-electrode arrays (Blackrock Microsystems, Salt Lake City, UT) were inserted through the dura into two DRGs (Monkey C: C6 and C7; Monkey T: C7 and C8) on the right side using a high-velocity inserter (Rousche and Normann, 1992). Reference wires were placed over the dura. After inserting the arrays, a connector was positioned over the laminectomy and cemented in place with dental acrylic. Before recovering from anesthesia, neostigmine bromide (Vagostigmin; 0.1 mg/kg; Shionogi, Osaka, Japan) was administrated to recover from the paralyzing effects of pancuronium bromide. Dexamethasone (Decadron; 0.82 mg; MSD, Tokyo, Japan), atropine (0.25 mg; Mitsubishi Tanabe Pharma, Osaka, Japan), and penicillin (penicillin G potassium; 50000 units; Meiji Seika Pharma, Tokyo, Japan) were administered preoperatively, and penicillin (50000 units) and ketoprofen (Capisten; 5 mg/kg; Kissei pharmaceutical, Matsumoto, Japan) were given postoperatively.

### Neural recording and spike detection

The implanted arrays consisted of 48 platinized-tip silicon electrodes (100–1000 kΩ at 1 kHz), arranged in a square grid (400 μm pitch) with 1 mm in length, and in a 5 × 10-configuration. The size of the array (2 mm in width, 4 mm in length) covered a DRG of 2–3 mm in diameter and 4 mm in length. For Monkey C, the electrode arrays were connected to a 96-channel amplifier (Plexon MAP system; Plexon, Dallas, TX) with a gain of ×20000, and signals from each electrode were sampled at 40 kHz. Filtered spikes (150–8000 Hz) above the amplitude threshold, which was determined by the “auto threshold algorithm” of the software, were bracketed within a window running 0.3 ms before to 0.8 ms after the threshold crossing. For Monkey T, the electrodes were connected to a 128-channel amplifier (Cerebus; Blackrock Microsystems, Salt Lake City, UT) with a gain of ×1000, and signals from each electrode were sampled at 30 kHz. Filtered waves (250–7500 Hz) above the amplitude threshold, which was 5 times the estimated background noise based on the median of the absolute value of the bandpass filtered signals (Quiroga et al., 2004), were captured within a window running 0.33 ms before to 0.73 ms after threshold crossing. After the detection of signals crossing the threshold in both monkeys, spikes with similar features on the principal component analysis (PCA) projection were grouped into clusters by semi-automatic spike sorting methods (Offline sorter; Plexon, Dallas, TX) and further manual refinement. The interval between 2 consecutive spikes was more than 1 ms for 51 units, which implied no contamination from other neurons. For the remaining 32 units, the proportion of cases in which the interval between two consecutive spikes was less than 1 ms was less than 1% of the total number of spikes. The neuronal firing rates for each unit were computed in 5-ms bins in synch with the sampling rate of the motion capture system at 200 fps.

### Classification procedure

We identified the modality of some recorded units each day in Monkey C right after the tasks was completed. Units that were sorted using an online spike sorting method were analyzed. Waves above the amplitude threshold, which was used in the above offline sorting, were sorted to the same units by selecting waveforms that crossed time-amplitude windows that were set manually. We considered that spikes constituting online sorted units were similar to, but not coincident with those constituting offline sorted units. The arm, hand, and fingers were stimulated manually to identify the receptive field of each unit. Pressure over the tendon and muscle belly was used to identify tendon organs and muscle spindles, respectively. Brushing and pinching were used to identify cutaneous receptors. Subtypes of cutaneous receptors were classified into rapidly or slowly adapting units based on their response to the stimuli.

### Motion capture

**Figure 1:**
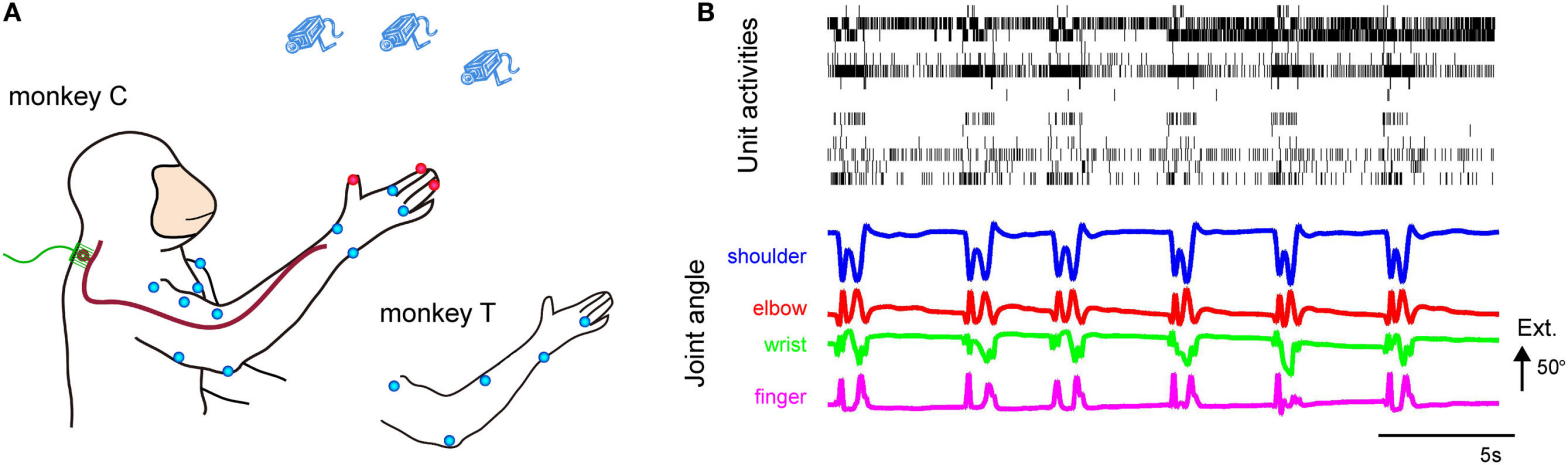
**(A)** Experimental setup for the simultaneous recording of DRG neuronal ensemble activity and forelimb joint kinematics from behaving monkeys. Microelectrode arrays were implanted in the right DRGs. Markers were placed on both shoulders and the right arm and hand. The blue dots represent 6-mm-diameter spheroid markers, and the red dots represent 4-mm-diameter spheroid markers. **(B)** Simultaneous recording of DRG neuronal ensemble activity and forelimb joint kinematics of Monkey C. (Top) Activity of 15 simultaneously recorded units in the C6 and C7 DRGs during reach-to-grasp movements of the right hand. (Bottom) Shoulder, elbow, wrist, and finger (digit 2 MCP) joint angles. Extension (Ext.) is represented by an upward deflection (arrow) of the traces; the length of the arrow represents the magnitude of the angle.

By tracking multiple reflective markers (4- or 6-mm-diameter spheroids) with an optical motion capture system (Eagle-4 Digital RealTime System; Motion Analysis, Santa Rosa, CA), movements of the upper limb, from the shoulder to the fingers, were recorded and synchronized with the neural recordings. In the system, 12 or 11 infrared cameras operated at 200 frames/s to track the position of the reflective markers with submillimeter accuracy in Monkey C and T, respectively. A total of 14 or 5 markers were attached to the surface of the forelimb using a mild adhesive (LACE FX TAPE; Vapon Inc., Fairfield, NJ, Aron Alpha Extra Jelly, Toagosei, Tokyo, Japan) in Monkey C and T, respectively (Figure 1A). For Monkey C, a total of 14 markers were attached to the left shoulder (marker 1; M1), the center of the chest (M2), the right shoulder (M3), the biceps (M4), the triceps (M5), the medial epicondyle (M6), the medial to the medial epicondyle (M7), the radial styloid process (M8), the ulnar styloid process (M9), the metacarpophalangeal joint of digit 2 (M10), the metacarpophalangeal joint of digit 4 (M11), the middle phalanx of digit 1 (M12), the middle phalanx of digit 2 (M13), and the middle phalanx of digit 4 (M14). For Monkey T, a total of 5 markers were attached to the right shoulder (M1), the medial epicondyle (M2), the center of the forearm (M3), the ulnar styloid process (M4), and the metacarpophalangeal joint of digit 4 (M5). The markers were placed at almost identical positions on the two recording days for Monkey C. The inter-marker distance between any two markers was similar between the 2 days. The ratio of the distance between the 2 days was 0.998 in the breast (M1–M2, M1–M3, and M2–M3), 1.01 in the upper arm (M1–M7), 1.03 in the lower arm (M7–M8 and M7–M9), 1.01 in the hand (M8–M9, M8–M10, M8–M11, and M10–M11), and 1.09 in the fingers (M8–M12, M10–M13, and M11–M14). A comprehensive catalog of 10 or 4 anatomically defined upper extremity joint angles (Monkey C: shoulder flexion/extension (FE), shoulder adduction/abduction (AA), elbow FE, pronation/supination (PS), wrist FE, wrist radial/ulnar (RU), digit1 FE, digit1 AA, digit2 FE, and digit4 FE; Monkey T: elbow FE, PS, wrist FE, and wrist RU) were analyzed in Monkey C and T, respectively (Monkey C, Table 1; Monkey T, Table 2). In particular, Euler angles were used to represent relative joint rotation. To reduce noise from various sources, temporal changes in the joint angles were smoothed using a 5 Hz low-pass digital filter. For convenience, we refer to the first and second time derivatives of the joint angles as “velocity” and “acceleration,” respectively.

**Table 1:** **Calculation of the joint angles in Monkey C. Joint angles were calculated from the two vectors presented in the right columns**.

**Joint angle**	**Two vectors**	
Shoulder FE	Cross product of vector M3–M2 and vector M3–M1	Vector M3–M7
Shoulder AA	Vector M3–M1	Vector M3–M7
Elbow FE	Vector M7–M3	Vector M7–M8/M9
PS	Projection of vector M7–M3 on the plane with the normal vector M7–M8/M9	Projection of vector M8–M9 on the same plane
Wrist FE	Cross product of vector M8–M10 and vector M8–M9	Vector M8/M9–M7
Wrist RU	Vector M9–M8	Vector M8/M9–M10
Digit1 FE	Projection of vector M8–M10 on the plane including M8, M10, and M11	Projection of vector M8–M12 on the same plane
Digit1 AA	Projection of vector M8–M10 on the plane with the normal vector M11–M10	Projection of vector M8–M12 on the same plane
Digit2 FE	Cross product of vector M8–M10 and vector M8–M11	Vector M10–M13
Digit4 FE	Cross product of vector M8–M10 and vector M8–M11	Vector M11–M14

**Table 2:** **Calculation of the joint angles in Monkey T. Joint angles were calculated from the two vectors presented in the right columns**.

**Joint angle**	**Two vectors**	
Elbow FE	Vector M2–M1	Vector M2–M4
PS	Projection of vector M2–M1 on the plane with the normal vector M2–M4	Projection of vector M3–M4 on the same plane
Wrist FE	Cross product of vector M4–M2 and vector M4–M3	Vector M5–M4
Wrist RU	Vector M4–M3	Vector M4–M5

### Encoding of joint kinematics

Joint angle, velocity, and acceleration were modeled as a weighted linear combination of neuronal activity using multidimensional linear regression analysis as follows:

(1) $$y_{j}(t)=\sum_{k,l}w_{j,k,l}\times x_{k}\left(t+l\delta \right)+b_{j}$$

where: *y*_*j*_(*t*) is a vector of kinematic variables *j* (joint angle, velocity, and acceleration) at time index *t*. *x*_*k*_(*t* + *l*δ) is an input vector of unit *k* at time index *t* and time-lag *l*δ (δ = 5 ms). *w*_*j,k,l*_ is a vector of weights on unit *k* at time-lag *l*δ, and *b*_*j*_ is a vector of bias terms to *y*_*j*_. The units that showed no more than one spike in the training data sets were omitted before the regression analysis. We used a Bayesian SLiR algorithm that introduced the automatic relevance determination (ARD) parameters α_*j,k*_ for the weights *w*_*j,k,l*_ assuming the same ARD parameters α_*j,k*_ for different time-lags *l*δ. Namely, sparse conditions were imposed only for the unit dimension, and not for the temporal dimension (Toda et al., 2011). By applying the variational Bayesian approximation (Sato, 2001), this method iteratively estimates the weights and the ARD parameters, which represent how much the weight contributes to the reconstruction. On the basis of the values of the estimated ARD parameters, the SLiR algorithm automatically and effectively selects important feature sets and prunes inappropriate signals from explanatory variables to attain a better generalized performance compared to the regularized linear model (Figueiredo and Nowak, 2003; Ganesh et al., 2008; Umeda et al., 2012). This is because having too many parameters relative to a limited number of training data sets is known to lead to poor generalized performance (Akaike, 1974; Geman et al., 1992). As external stimulation induced afferent activity, the time-lag was set at future, positive values. The recorded neuronal population consisted of different types of sensory neurons. Even muscle spindle discharges are determined not only by the current kinematic state of their parent muscles but also by the simultaneous activation of the fusimotor systems during active movements. Thus, it is difficult to determine the optimum value of the time window during the ensemble coding based on physiological knowledge elicited from previous experiments using single afferent recordings. In this study, we used a time window in which maximum accuracy was achieved for the estimation of joint kinematics. When we changed the length of the time window, 400 and 150 ms were obtained in Monkey C and T, respectively (data not shown). If we consider the conduction velocity of afferent nerves to be more than 10 m/s (Loeb, 1976) and their length to be ~30 cm, the propagation delay should be less than 30 ms. One possible explanation for such long windows is that good prediction of the encoding of joint kinematics for 3-dimensional movements requires sufficient amounts of DRG activity, but the firing frequency of individual DRG neurons is quite low.

To examine what timing of DRG neuronal activity encoded joint kinematics, joint kinematics were modeled as a weighted linear combination of activity of DRG neurons using the SLiR algorithm. In the analysis, the model was generated from the spike number in a fixed time window (25 ms; 5 bins) at a variable time-lag relative to joint kinematics. We assessed each model generated from input-output pairs with a different time-lag by calculating the correlation coefficient between the observed kinematics and its prediction.

Since the available data volume was limited in the encoding of joint kinematics by reconstructed DRG neuronal activity (Monkey C, 3 blocks; Monkey T, 1 block, result shown in Figure 7), the models generated with the data sets were assessed using the same data sets. When we changed the length of the time window in the analysis, maximum accuracy was achieved for the estimation of joint kinematics at approximately 150 and 500 ms in Monkey C and T, respectively (data not shown). We used this time window in the analysis.

### Decoding of spike timing

Neuronal firing frequency (dimensions; per second) was generated by calculating the inverse of the inter-spike-interval of each unit. The values were assigned to 5-ms bins corresponding to the intervals. The firing frequency of the DRG neuronal ensembles was modeled as a weighted linear combination of joint kinematics (joint angle, velocity, and acceleration) using the following multidimensional linear regression algorithm:

(2) $$x_{j}(t)=\sum_{k,l}w_{j,k,l}\times y_{k}\left(t+l\delta \right)+b_{j}$$

where: *x*_*j*_(*t*) is a vector of the firing frequency of unit *j* at time index *t*. *y*_*k*_(*t* + *l*δ) is an input vector of kinematic variables *k* at time index *t* and time-lag *l*δ (δ = 5 ms). *w*_*j,k,l*_ is a vector of weights on kinematic variables *k* at time-lag *l*δ, and *b*_*j*_ is a vector of bias terms to *x*_*j*_. We used a Bayesian SLiR algorithm that introduced sparse conditions for the kinematic dimension. As external stimulation was the cause of afferent activity, the time-lag was set at past, negative values. When we varied the length of the time window, maximum accuracy of estimation of the joint kinematics was achieved at −150 ms (data not shown). We used this time window when decoding neuronal firing frequency from forelimb kinematics.

To acquire the spike timing of the DRG neuronal ensembles from their firing frequency, the inverse operation was employed. Firing frequency values in 5-ms bins were summed cumulatively until they hit a constant threshold of 200. The threshold value, 200, was set because the firing frequency values (dimension; per second) were assigned to 5-ms bins. At this time, a spike occurred and the cumulative sum was reset to zero. Then, the integration process started again. When the firing frequency is replaced with the membrane potential, the procedure corresponds to the ordinary IF model.

### Data analysis

In both the encoding of joint kinematics by the population activity of DRG neurons and the estimation of firing frequency from the kinematics, a model generated from the training data set was tested against a test data set. For Monkey C, continuously recorded data of each session were partitioned into 24 blocks (1 block for 25 s data). Among the 24 blocks, 21 randomly selected blocks were used as the training data set, and the remaining 3 blocks as the test data. For Monkey T, continuously recorded data were partitioned into 6 blocks (1 block for 12.5 s data). Among the 6 blocks, 5 randomly selected blocks were used as the training data set, and the remaining block was used as the test data. To assess the ability of the model encoding joint kinematics, a 5- and 6-fold cross-validation was performed in Monkey C and T, respectively. To validate the prediction power of the model, we created surrogate training data sets in which the temporal firing profiles of inputs were shuffled independently across different trials and tested subsequently for their prediction of each output parameter.

To assess the fine structures of the spike pattern, cross correlation between the observed firing pattern and its prediction in the test data sets was calculated. Then, an absolute value of time-lag at which the maximum correlation was achieved was obtained.

### Statistical analysis

The data were analyzed using the Kolmogorov-Smirnov test, a One-Way analysis of variance (ANOVA), or the non-directional paired Student's *t*-test, with Bonferroni correction if necessary. An alpha level of significance was set at 0.05 for all statistical tests. We found 95% confidence intervals for proportions based on the inverse of the appropriate cumulative Beta distribution. We used MATLAB (MathWorks, Natick, MA) for all statistical analyses.

## Results

### Simultaneous recording of ensemble activity of DRG neurons

We recorded neuronal activity from the DRGs at the C6/7 and C7/8 segments with two multi-electrode arrays in Monkey C and T, respectively. For Monkey C, a couple of units could still be recorded by the fifth post-operative day. We used recording data in 3 sessions on the third post-operative day and 2 sessions on the fourth post-operative day for further analysis. A total of 15 or 16 units were discriminated from 13 channels on the third post-operative day, and a total of 12 units were discriminated from 9 to 10 channels on the fourth post-operative day. The recorded units included muscle spindles, cutaneous receptors (both rapidly and slowly adapting units), and a tendon organ in Monkey C. In Monkey T, a total of 13 units were discriminated from 12 channels in one session on the second post-operative day. After the recording, due to accidental damage to the head holder, further recordings could not be performed.

### Reconstruction of forelimb joint kinematics

Population recordings during voluntary reach-to-grasp movements showed that the temporal discharge patterns of individual isolated units were correlated with temporal changes in the joint angles and that the temporal firing pattern of each unit was different among the isolated units (Figure 1B).

**Figure 2:**
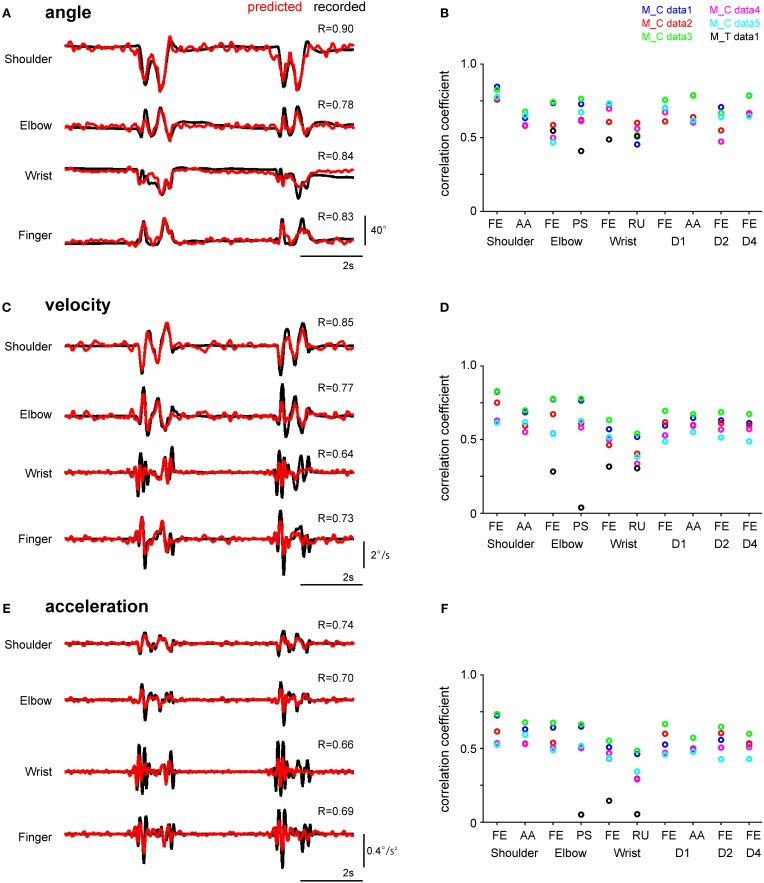
**Performance of the SLiR model in predicting joint kinematics from DRG ensemble activity during reach-to-grasp movements. (A,C,E)** Examples of recorded kinematics of the shoulder, elbow, wrist, and finger (digit 2 MCP) joints of Monkey C (black line) and their prediction using the SLiR model (red line). The correlation coefficient (R) between the recorded and predicted kinematics is shown in the upper right corner of each trace. The angular changes at the joints are shown in **(A)**, velocity in **(C)**, and acceleration in **(E)**. **(B,D,F)** Test performance (correlation coefficient: R) of the SLiR model in predicting the kinematics of the forelimb joints. The indicated values are averages of the results of 5 or 6 pairs of training and test data sets from each session. The angular changes at the joints are shown in **(B)**, velocity in **(D)**, and acceleration in **(F)**. FE, flexion/extension; AA, adduction/abduction; PS, pronation/supination; RU, radial/ulnar.

To investigate whether the neuronal activity in the DRGs conveyed information about joint kinematics, we applied the SLiR model to encoding of kinematic variables using the activity of all units. As the excitation of peripheral afferents can be elicited by external stimulation, we considered that the peripheral afferents contained information concerning limb position immediately before firing (Umeda et al., 2012). Therefore, the kinematic variables were defined as a weighted sum of neural activity for the upcoming 400 and 150 ms (here grouped into 80 and 30, 5-ms bins) in the SLiR algorithm in Monkey C and T, respectively. Figure 2 shows the results for the reconstruction of angle (Figures 2A,B), velocity (Figures 2C,D), and acceleration (Figures 2E,F) of forelimb joints in a test data set, from the activity of a neuronal ensemble. The prediction accuracy of 3 kinematics of all 10 joints encoded by the actual neural firing pattern was much better than that obtained by the shuffled data (paired Student's *t*-test, *p* < 0.0001). Thus, the SLiR model provided accurate predictions of joint kinematics. The prediction accuracy for angle was higher than that for velocity and acceleration in all joints (One-Way ANOVA, *F*_(2, 2319)_ = 254.03, *p* = 1.76 × 10^−100^; paired Student's *t*-test with Bonferroni correction (*n* = 3), *p* < 0.0001). These results demonstrate that a population of DRG neurons convey rich information about joint kinematics, especially for angle.

**Figure 3:**
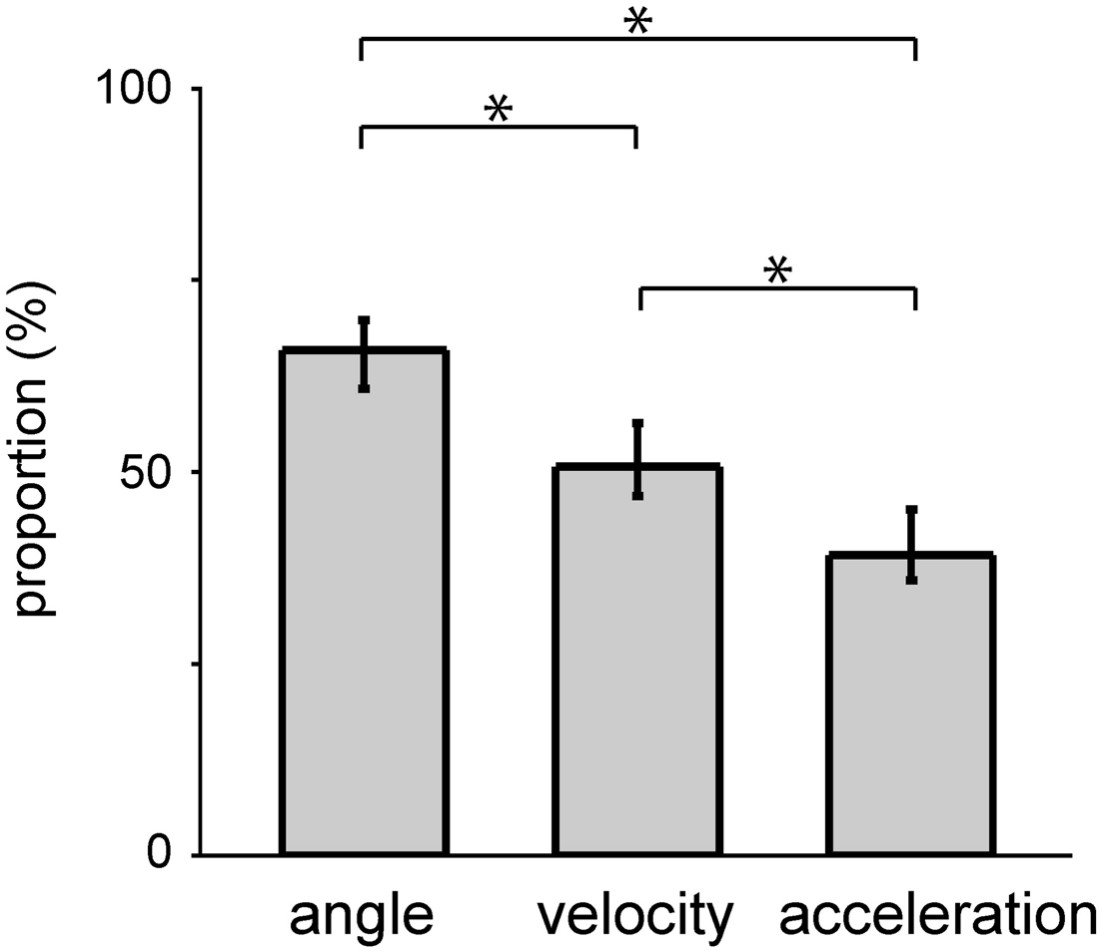
**Selection of a subset of DRG neurons of two monkeys by the SLiR model**. The proportion of units selected by the SLiR model during the encoding of the angle, velocity, and acceleration of the forelimb joints are shown. The asterisks indicate that there was a significant difference between two groups. The error bars represent the confidence intervals for the proportions.

The SLiR model reduced the number of units used in the prediction (Figure 3). Note that the proportion of selected units was higher in the prediction of angle than that of velocity and acceleration [angle, 0.66 (0.61–0.70); mean, (confidence interval)]; velocity, 0.52 (0.47–0.56); acceleration, 0.41 (0.36–0.45); One-Way ANOVA, *F*_(2, 90)_ = 22.34, *p* = 1.32 × 10^−8^; paired Student's *t*-test with Bonferroni correction (*n* = 3), *p* < 0.0001), suggesting that a larger number of units contribute to the encoding of joint angle than velocity and acceleration. This result was correlated with the prediction accuracy of the respective kinematics shown in Figure 2. In any case, the SLiR model accurately encoded temporal changes in joint kinematics from the activity of the DRG neuronal ensembles using a reduced number of units.

**Figure 4:**
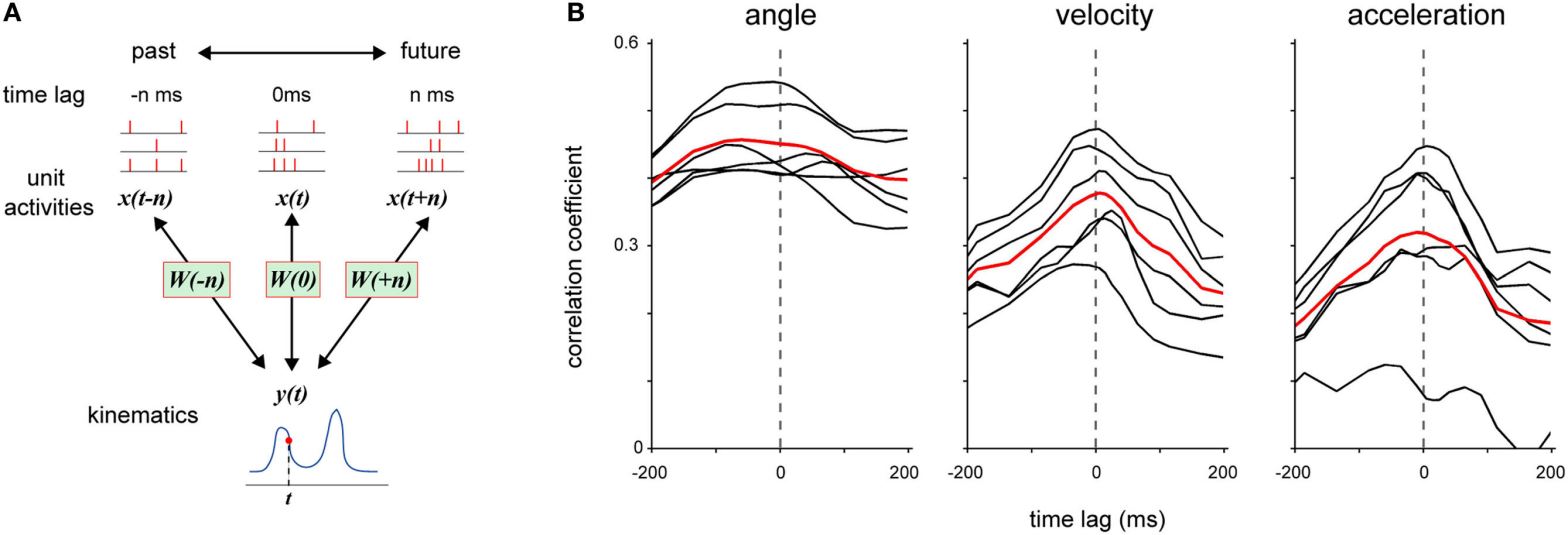
**Examination of timing of DRG neuronal ensemble activity that contributed to the encoding of forelimb joint kinematics. (A)** Each model *W* was fitted to DRG neuronal ensemble activity *x* in a 25-ms time window at various time-lag *n* relative to the kinematics *y*. When time-lag *n* was a positive value, the model *W* was fitted to DRG neuronal ensemble activity *x*(*t* + *n*), which was detected at *n* ms after the kinematics *y(t)*. **(B)** Prediction performance (correlation coefficient) of each model that was fitted to DRG neuronal ensemble activity at various time-lags relative to joint angle, velocity, and acceleration, respectively. The black lines represent the average results in individual sessions and the red lines represent the average results in all sessions for both monkeys.

Next, we examined how the timing of neuronal activity encoded the joint kinematic information. We generated models from neuronal activity in a short time window (25 ms) with variable time-lag against joint kinematics and assessed each model by calculating the correlation coefficient between the observed kinematics and its prediction (Figure 4A). The activity of DRG neuronal ensembles with similar timing as the kinematics provided the most accurate prediction of all three measures of kinematics (Figure 4B). Neuronal activity for 375 ± 44.3 ms (mean ± SE, *n* = 6) reconstructed a joint angle at 80% of the highest accuracy. On the other hand, neuronal activity for 150 ± 15.8 ms and 150 ± 9.1 ms reconstructed the velocity and acceleration at the highest accuracy of 80%, respectively. The range for the prediction of a joint angle was significantly larger than that of velocity and acceleration (One-Way ANOVA, *F*_(2, 15)_ = 22.09, *p* = 3.38 × 10^−5^; paired Student's *t*-test with Bonferroni correction (*n* = 3), *p* < 0.05).

### Reconstruction of the firing frequency of DRG neurons

**Figure 5:**
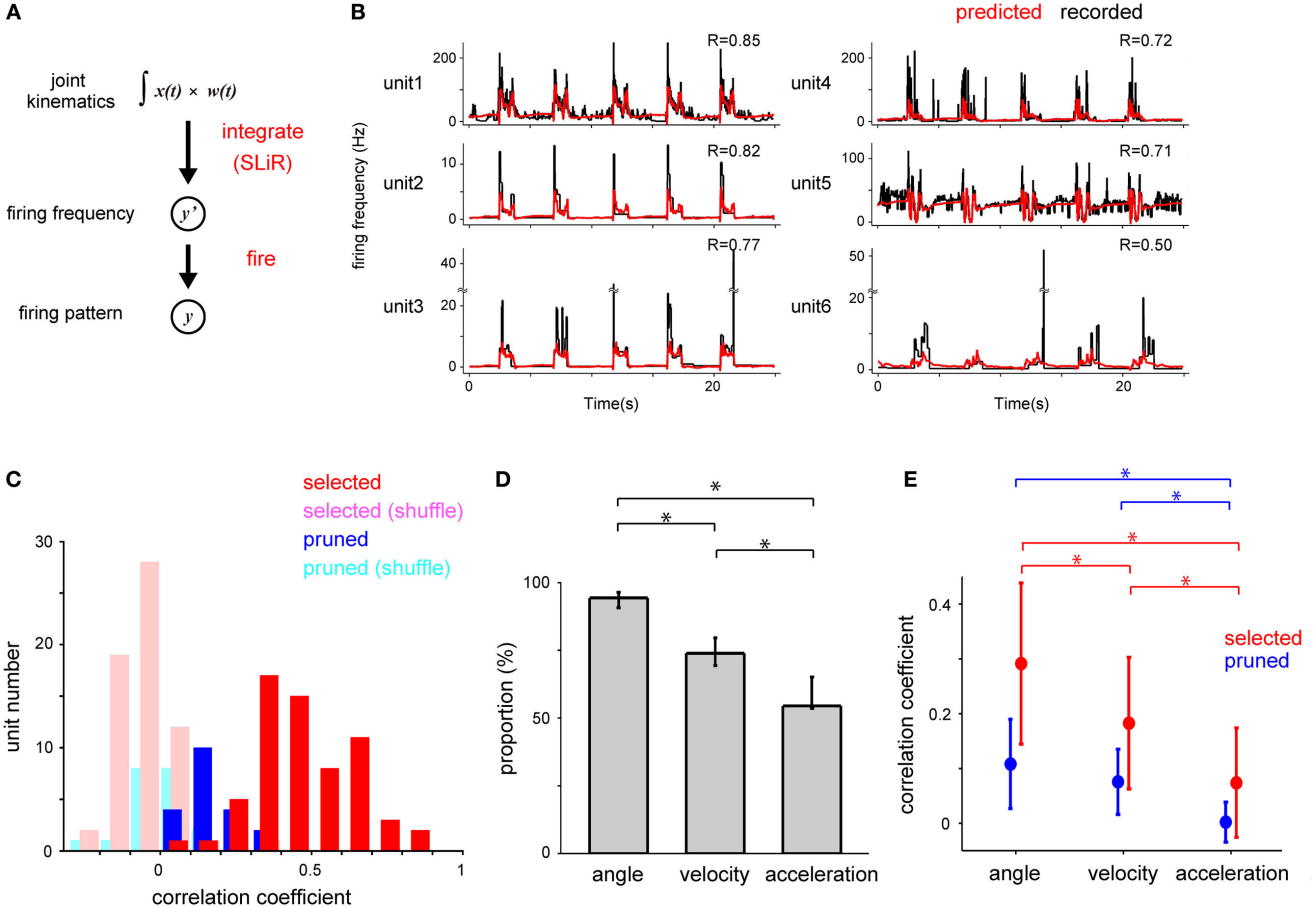
**Decoding of the temporal change of firing frequency by forelimb joint kinematics. (A)** Schematic drawing of the estimation of DRG neuronal ensemble activity from the forelimb joint kinematics using a serial combination of the SLiR algorithm and a fire model. **(B)** Examples of recorded temporal changes of the firing frequency of 6 neurons in Monkey C (black line) and their prediction using the SLiR model (red line). The correlation coefficient (R) between the recorded and predicted firing frequency is shown in the insets. **(C)** Histograms of the correlation coefficient between the observed firing frequency and the prediction by the SLiR. The histograms are shown for the units selected in the encoding of forelimb joint kinematics by the SLiR model (red bar) and the pruned units (blue bar). The light red and blue bars represent the histogram for the selected and pruned units in the shuffled kinematic data, respectively. The total number of selected and pruned units were 63 and 20, respectively. **(D)** Selection of a subset of kinematic variables from both monkeys by the SLiR model. The proportion of kinematic variables selected by the SLiR model in the encoding of firing frequency of DRG neurons is shown. The asterisks indicate significance. The error bars represent the confidence intervals for the proportions. **(E)** Contribution of angle, velocity, and acceleration components of kinematics to the reconstruction of the firing frequency. Reconstruction was conducted from each kinematic component and weighted values determined by the SLiR model with population data. Graphs are shown for the selected (red) and pruned units (blue). The indicated values are the average results in 6 sessions. The asterisks indicate significance. The error bars represent the standard deviation of the mean.

We estimated the temporal firing pattern of DRG neuronal ensembles using an IF model. We performed the calculation in two steps (Figure 5A). For the first step (integration process), we applied the SLiR model to estimate the firing frequency of DRG neuronal ensembles from all kinematic variables. We then calculated the spike timing of individual units from the decoded firing frequency as the second step (fire process). In the fire process, the firing frequency values were summed cumulatively until a constant threshold was reached. At this time, a spike occurred and the cumulative sum was reset to zero, and integration started again.

We considered that peripheral afferents carried information concerning limb position within time windows preceding the spiking events. Then, we defined firing frequency as a weighted sum of joint kinematics for the previous 150 ms (here grouped into 30, 5-ms bins) in the SLiR model. Figure 5B shows the results of the estimation of firing frequency of 6 units in a test data set from the joint kinematics in Monkey C. In the previous analysis in Figure 3, the SLiR model selected important units that contributed to the encoding of joint kinematics and pruned irrelevant units (selected, 63 units; pruned, 20 units). For 61.9% of the selected units, the prediction accuracy (correlation coefficient) was more than 0.4 (Figure 5C). The prediction accuracy from the actual joint kinematics was much better than that from the shuffled data in 95.2% of the selected units and 65% of the pruned units (paired Student's *t*-test, *p* < 0.05; Figure 5C). The prediction accuracy of the selected units was higher than that of the pruned units (selected units, 0.47 ± 0.02 (mean ± SE); pruned units, 0.17 ± 0.02; Student's *t*-test, *p* < 0.0001; Figure 5C). The SLiR model reduced the number of inputs used in the prediction (Figure 5D). Note that the proportion of the selected angle was higher than that of velocity and acceleration in the prediction of the firing frequency [angle, 0.94 (0.91–0.96); mean, (confidence interval)]; velocity, 0.74 (0.69–0.80); acceleration, 0.55 (0.54–0.65); One-Way ANOVA, *F*_(2, 90)_ = 42.6, *p* = 9.56 × 10^−14^; paired Student's *t*-test with Bonferroni correction (*n* = 3), (*p* < 0.05), suggesting that a higher number of angle variables contributed to the encoding of the firing frequency than those of velocity and acceleration. These results show that the kinematic information of forelimb joints, especially joint angles, can be translated to the firing frequency of DRG neurons using the SLiR model.

To demonstrate the importance of individual kinematic variables in the reconstruction of the firing frequency, we calculated the correlation coefficient between the observed firing frequency and the predictions derived from each kinematic and the corresponding weight values determined through the SLiR model among all the kinematics. The joint angle contributed most to the decoding of the firing frequency of the selected units (angle, 0.29 ± 0.02 (mean ± SE, *n* = 63); velocity, 0.18 ± 0.02; acceleration, 0.07 ± 0.01; One-Way ANOVA, *F*_(2, 186)_ = 47.83, *p* = 1.74 × 10^−17^; paired Student's *t*-test with Bonferroni correction (*n* = 3), *p* < 0.05; Figure 5E), but not of the pruned units. This result agrees with the preceding analysis that the activity of the DRG neuronal ensembles encoded the joint angle more accurately than the velocity and acceleration of the joints (Figure 2).

### Reconstruction of the spike timing of DRG neurons

**Figure 6:**
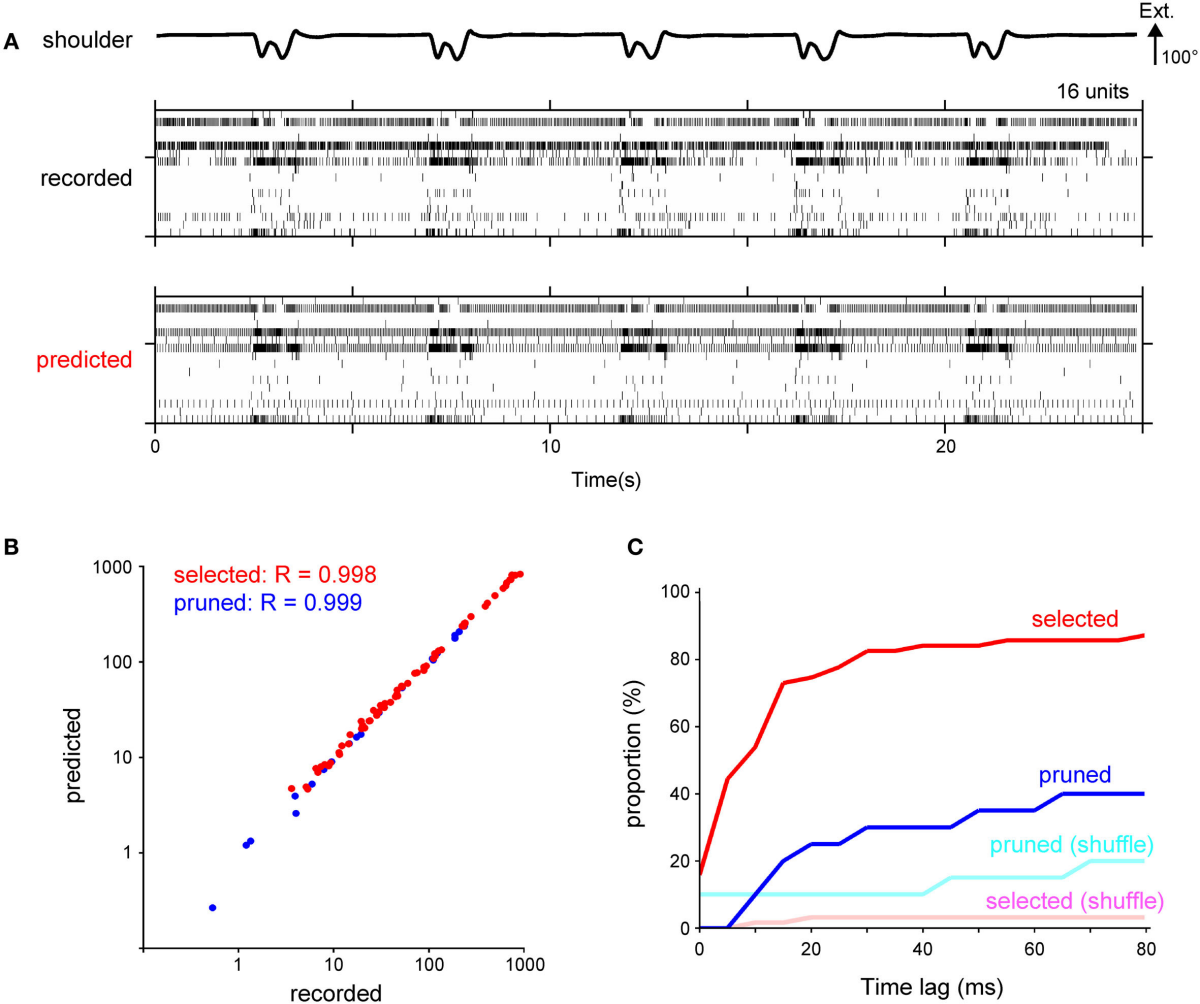
**Calculation of spike timing from the temporal change of the firing frequency using a fire model. (A)** Raster plot of recorded spikes from 16 neurons in Monkey C (middle) and their prediction (bottom). Shoulder joint angle is shown in the top trace. Extension (Ext.) is represented by an upward deflection (arrow) of the traces; the length of the arrow represents the magnitude of the angle. **(B)** A logarithmic scatter plot shows the relationship between the total number of recorded and predicted spikes. Each point represents a unit selected in the encoding of the forelimb joint kinematics by the SLiR model (red) or a pruned unit (blue). The correlation coefficient (R) between the number of the recorded and predicted spikes is shown in the upper left corner of the graph. **(C)** Cumulative frequency histogram of time-lags that were determined by cross-correlations between the observed and predicted firing pattern. Histograms are shown for the units selected in the encoding of the forelimb joint kinematics by the SLiR model (red) and the pruned units (blue). The light red and blue bars represent the histogram of the time-lag between the observed firing pattern and the prediction from the shuffled kinematic data for the selected and pruned units, respectively.

Next, we calculated the spike timing of individual DRG neurons from the decoded firing frequency. Figure 6A shows raster plots for 16 units in a test data set of an actual recording and its prediction. The overall firing pattern in the prediction was similar to that in the recorded data. To assess the general structure of the estimated firing pattern, we compared the total number of spikes between the recorded and predicted data. For 89% of the selected units and 95% of the pruned units, there was no significant difference in the total number of spikes. The correlation coefficient for the total number of spikes was 0.998 and 0.999 for the selected and pruned units, respectively (Figure 6B). Next, we examined the fine structure of the predicted firing pattern by calculating the cross-correlation for the binned spike number of the recorded and predicted data. A time-lag, which resulted in a maximum cross-correlation value, was less than 10 ms for 54% of the selected units and less than 20 ms for 75% of the selected units (Figure 6C). The time-lag for the selected units was smaller than the time-lag derived from a firing pattern decoded by the shuffled kinematics [recorded data, 10 ms (median, *n* = 63); shuffled data, 1245 ms; Kolmogorov-Smirnov Test, *p* < 0.05; Figure 6C], but not for the pruned units [recorded, 2135 ms (median, *n* = 20); shuffled, 1605 ms; Kolmogorov-Smirnov Test, *p* = 0.28; Figure 6C]. Thus, the IF model incorporating the SLiR algorithm provided accurate decoding of the spike timing of the DRG neuronal ensembles from the joint kinematics, especially for the selected units.

**Figure 7:**
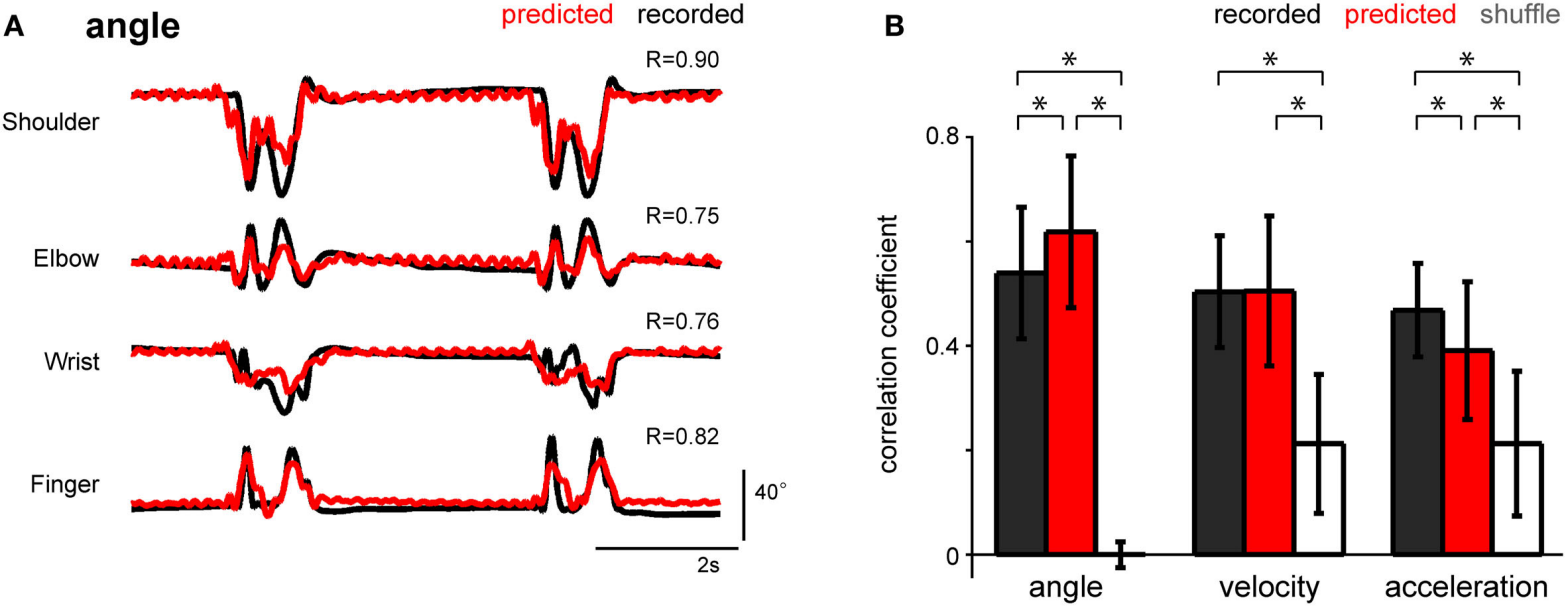
**Performance of the SLiR model in predicting joint kinematics from the predicted DRG neuronal ensemble activity. (A)** Examples of recorded kinematics from the shoulder, elbow, wrist, and finger (digit 2 MCP) joints (black) and their prediction from DRG activity using the SLiR model (red). The correlation coefficient (R) between the recorded and predicted kinematics is shown in the upper right corner of each graph. **(B)** Test performance [correlation coefficient (R)] of the SLiR model in predicting the kinematics of the forelimb joints from the recorded (black), predicted (red), and shuffled (white) activity. The indicated values are the average results from 6 sessions and the error bars represent the standard deviation (*n* = 274 data sets). The asterisks indicate significance [paired Student's *t*-test with Bonferroni correction (*n* = 3), *p* < 0.0001].

To determine the optimal parameters of afferent electrical stimulation to transmit proprioceptive signals from a neuroprosthesis, the stimulus timing should be able to encode the kinematic information. We applied the SLiR model to the reconstruction of kinematic variables from the estimated spike timing. Figure 7A shows the reconstruction of the angle of 4 forelimb joints from the predicted spike timing of a neuronal ensemble. The prediction performance of all 3 kinematic variables calculated by using the estimated spike timing was much better than that calculated by using the shuffled spike data (paired Student's *t*-test with Bonferroni correction (*n* = 3), *p* < 0.0001; Figure 7B). Furthermore, the prediction accuracy of the joint angle calculated by using the estimated neuronal firing pattern was higher than that calculated by using the recorded neuronal firing (paired Student's *t*-test with Bonferroni correction (*n* = 3), *p* < 0.0001; Figure 7B). Thus, the estimated multi-unit activity encoded the joint kinematics, suggesting that the model used to estimate the spike timing of individual units from the joint kinematics can be useful for the design of stimulus parameters that provide proprioceptive information from the periphery.

## Discussion

An ideal proprioceptive neural interface should enable individuals to perceive proprioception that is driven by electrical stimulation of the nervous system as if it comes from their own body. Vibratory or electrical stimulus of a tendon excites muscle spindles or cutaneous receptors to produce the illusion of movement in humans (Goodwin et al., 1972; Craske, 1977; McCloskey et al., 1983; Gandevia, 1985; Collins and Prochazka, 1996; Dhillon and Horch, 2005; Roll et al., 2009; Thyrion and Roll, 2010). Simulated patterns of vibration to a couple of tendons have been shown to induce illusory multi-joint movements (Thyrion and Roll, 2010). Thus, it is important to optimize the parameters of peripheral electrical stimulation that produces kinesthetic illusion as a part of a somatosensory BMI. One strategy for the design of optimal stimulation patterns to afferents is to mimic the neuronal representation of limb positions and kinematics. At an early stage of the development of BMI, a decoder was designed based on experimental evidence for the causal relationship between the activity of neurons in the primary motor cortex and kinematic parameters of limb movements or muscle activity (Chapin et al., 1999; Wessberg et al., 2000; Serruya et al., 2002; Morrow and Miller, 2003). As an analogous strategy, understanding decoding rules in which limb movements are transformed into the activity of sensory neurons is an important first step toward a proprioceptive neural interface. We showed that an IF model incorporating the SLiR algorithm accurately predicted the electrical activity patterns of DRG neuronal ensembles from forelimb joint kinematics. The predicted spike patterns contained kinematic information of forelimb joints at a similar extent to the recorded neural activity. These results suggest that the decoding method used in this study will facilitate the optimization of electrical stimulation parameters for the production of artificial kinesthetic sensation.

### Multichannel recording from the cervical DRGs of behaving primates

The activity of peripheral afferents has been recorded by single fiber recordings from humans and awake animals. These studies were able to identify the response properties of single afferent fibers to various external mechanical stimulation or both passive and voluntary movements (Hagbarth and Vallbo, 1967, 1968, 1969; Matthews, 1972; Gandevia and McCloskey, 1976; McCloskey, 1978; Loeb and Duysens, 1979; Schieber and Thach, 1985; Edin and Vallbo, 1990; Flament et al., 1992). The kinesthetic sense arises from the activity of a neuronal population (Gilhodes et al., 1986), but the previous single fiber recording studies demonstrated difficulty in describing decoding rules at the population level. In the present study, we recorded the population activity of peripheral afferents simultaneously from voluntarily behaving monkeys using multi-electrode arrays. The results demonstrate that temporal changes in the angle, velocity, and acceleration of various forelimb joints can be reconstructed from the activity of DRG neuronal ensembles, suggesting that the population activity of peripheral afferents encodes forelimb joint kinematics.

Previous multichannel recording studies of DRGs have been performed in anesthetized animals. These studies used multi-electrode arrays on cervical (Umeda et al., 2012) or lumbar (Stein et al., 2004; Weber et al., 2011) DRGs and detected neural signals from approximately 100 sensory neurons. Here, for the first time, we recorded simultaneously the activity of DRG neuronal ensembles using multi-electrode arrays from behaving non-human primates. The number of detected units in this study was fewer than in the previous recordings from anesthetized animals. Although the prediction accuracy for the encoding of joint kinematics from DRG population activity was lower than the previous experiments using anesthetized animals (Umeda et al., 2012), this study showed that joint kinematics could be reconstructed successfully from the activity of a fewer number of units at a certain level of accuracy (Figures 2, 3). Since muscle spindle discharge is affected by fusimotor drive, active movements may differ from the pattern of peripheral proprioceptive inputs that are merely generated by passive movements (Hunt and Kuffler, 1951; Matthews, 1972). The arm position is perceived more accurately during active movements than passive movements (Paillard and Brouchon, 1968; Bairstow and Laszlo, 1979; Gritsenko et al., 2007). Therefore, it is important to study the kinesthetic mechanism by analyzing neuronal activity in awake subjects rather than in anesthetized subjects, and proprioceptive signals arising from self-initiated movements rather than externally imposed movements.

Microneurography is a powerful technique and has provided much data concerning the physiological properties of primary afferents. However, microneurography is technically difficult to perform on subjects performing multi-joint movements, such as reaching and grasping movements, because an isolated afferent nerve is recorded by inserting a single fine electrode into the peripheral nerve of the moving limb (Vallbo et al., 2004). The activity of single DRG neurons has been recorded in awake monkeys (Schieber and Thach, 1985; Flament et al., 1992); however, they were only able to analyze the movement of the wrist in one direction. We were able to record afferent discharges during reaching movements of the entire forelimb for 10 min. Although the recording of individual units was stable during only 1 session, the shape of the units was not affected by movement of the forelimb. The amount of data was sufficient to assess the relationship between population activity in DRG neurons and joint kinematics. Recording stability requires further improvement of the chronic implantation of electrode arrays for a BMI with sensory feedback.

### Encoding of joint kinematics by the activity of DRG neuronal ensembles

We showed that the SLiR algorithm accurately predicted the kinematics of various forelimb joints, including not only proximal joints, but also distal joints using the same data set. The coding of positions and movements of the ankle joint by a population of peripheral afferents was examined by collecting a number of separate microneurographic recordings during repeated movements (Bergenheim et al., 2000; Roll et al., 2000; Jones et al., 2001; Aimonetti et al., 2007). However, these studies demonstrated that movement directions in two-dimensional space at a single joint of a leg could be estimated using the collected recording data. Although a reach-to-grasp movement seems to be a simple motion, it is a movement with multiple degrees of freedom. PCA of joint kinematics showed that 90% of the overall variability was accounted for by the first 12 principal components among a total of 30 principal components for Monkey C, and was accounted for by the first 7 principal components among a total of 12 principal components for Monkey T. The SLiR analysis in this study showed that the simultaneously recorded activity of DRG neuronal ensembles represents kinematic information of multiple joints of the forearm at multiple degrees of freedom.

The activity of the DRG neuronal ensembles encoded joint angle with higher correlations compared to velocity and acceleration (Figure 2). According to recent reports of microneurographic recordings from a single afferent in voluntarily moving human subjects, the activity of a single group Ia afferent from the muscle spindle encodes velocity and acceleration and that of a group II afferent conveys velocity information (Dimitriou and Edin, 2008a,b). In the present study, we recorded population activity containing the activity of cutaneous receptors that also contributed to the kinesthetic sense in addition to that of muscle spindles (Collins and Prochazka, 1996; Collins et al., 2005; Cordo et al., 2011). Hence, the summation of population activity from variable peripheral receptors may indicate that the best encoded kinematics is the joint angle. Population activity encoded angle information at 180 ms before spike discharge, and velocity and acceleration information at 75 ms before the spike discharge (Figure 4B). These results suggest that the kinematic information preceding to the movement is useful for accurate estimation of spike timing. This finding implies that a model that calculates stimulus timing from kinematic information of forelimb joints is practical for an online feedback system.

### An IF model incorporating the SLiR algorithm

The IF model has proven to be useful in addressing the question of how joint kinematics are encoded in the response of neurons. The leaky version of the IF model accurately predicted the spike timing of a peripheral sensory neuron in response to an external stimulus and succeeded in describing the functional properties of the receptor to this stimulus (Pillow et al., 2005; Kim et al., 2010; Dong et al., 2013). The model has been used to predict the responsiveness of single neurons. In the present study, we divided the model into two steps and fitted a model in the first step to data from a neuronal population that was recorded simultaneously from the DRGs. Through a fire process, the model provided an accurate prediction of spike timing at the population level.

During the integration of inputs, we used the SLiR algorithm. The SLiR algorithm automatically and effectively selected relevant feature sets from many parameters to attain a higher generalized performance than that obtained from other ordinary linear regression models (Figueiredo and Nowak, 2003; Ganesh et al., 2008). Its superior generalized performance was indicated previously using population recordings from the DRGs of anesthetized monkeys (Umeda et al., 2012). By selecting the optimal ensemble from joint kinematic variables for the decoding of the firing rate, the analysis revealed that joint angle contributed the most to the decoding of spike frequency. The results are consistent with those in the encoding analysis; the population activity of DRG neurons encoded the joint angle at the highest prediction accuracy.

In a somatosensory BMI, proprioceptive feedback should be applied to the nervous system in real time. For the model to be available practically for rapid proprioceptive feedback, the computer load should be reduced so as to conduct the calculations in real time. The reduction of input parameters without any deterioration of prediction performance may prove to be useful in BMI systems that limited hardware speed. The SLiR algorithm selected important kinematic information automatically from the entire recorded data without affecting model performance through machine learning. Therefore, the IF model incorporating the SLiR algorithm is a practical method for the application of rapid proprioceptive feedback to the brain in a bidirectional sensory-motor BMI.

### Limitations of the IF model incorporating the SLiR algorithm

Our results showed that the IF model incorporating the SLiR algorithm successfully reconstructed the temporal firing pattern of DRG neuronal ensembles from forelimb joint kinematics. However, the reconstruction deviated from the original firing pattern, which may have arisen from a smoothing effect introduced by the linear regression analysis that was used during the integration step. In the linear regression analysis, the output was obtained from a weighted sum of the inputs. This procedure potentially has a smoothing effect on the output. As shown in Figure 5B, the rapid increase of firing frequency could not be reconstructed by the SLiR algorithm, suggesting that the linear regression analysis failed to reproduce correctly the dynamic property of DRG neuronal activity. Second, it is difficult for the linear regression analysis to reproduce a flat baseline near zero when units do not fire (Figure 5B, unit6). Small constant values in the baseline can yield some spikes through the IF procedure where there was no firing in the original data. This phenomenon led to an increase of background activity in some units. If these deviations could be reduced, spike timing can be reproduced from the kinematics more accurately. In spite of the limitations of the model, its simplicity is a strong advantage for its use for generation of stimulus parameters in a proprioceptive interface.

### Application of a proprioceptive interface to a BMI

Peripheral electrical stimulation can activate various modalities of peripheral afferents including muscle spindle afferents, Golgi tendon organs, joint receptors, cutaneous receptors, and pain receptors. Information about limb position and movement is conveyed to the central nervous system via the activity of cutaneous receptors (Clark et al., 1979; Edin and Johansson, 1995; Edin, 2001; Cordo et al., 2011) as well as muscle spindles and joint receptors (Goodwin et al., 1972; Gandevia and McCloskey, 1976; Craske, 1977; McCloskey et al., 1983; Gandevia, 1985; Ferrell et al., 1987; Collins and Prochazka, 1996). Our previous study indicated that adding the neuronal activity of cutaneous receptor ensembles to that of muscle receptors including muscle spindles and joint receptors significantly improved the decoding accuracy of forelimb kinematics provided by the activity of muscle receptors only in anesthetized monkeys (Umeda et al., 2012). The recorded units in the present study included muscle spindles, cutaneous receptors, and a Golgi tendon organ in Monkey C, which encoded the forelimb joint kinematics. Thus, stimulation of a combination of muscle spindles, joint receptors, and cutaneous receptors may induce more realistic perception of limb sensation than stimulation of a single modality of peripheral afferents. Conversely, there is a possibility that peripheral stimulation can activate pain receptors and generate noxious sensations to subjects. Since the stimulation threshold of pain receptors is higher than that of muscle spindle afferents, joint receptors, and the majority of cutaneous receptors (Lloyd, 1943; Marchettini et al., 1990), control of stimulation strength can allow one to avoid generation of noxious sensation.

### Conflict of interest statement

The authors declare that the research was conducted in the absence of any commercial or financial relationships that could be construed as a potential conflict of interest.
